# Ribose Supplementation Alone or with Elevated Creatine Does Not Preserve High Energy Nucleotides or Cardiac Function in the Failing Mouse Heart

**DOI:** 10.1371/journal.pone.0066461

**Published:** 2013-06-18

**Authors:** Kiterie M. E. Faller, Debra J. Medway, Dunja Aksentijevic, Liam Sebag-Montefiore, Jürgen E. Schneider, Craig A. Lygate, Stefan Neubauer

**Affiliations:** Division of Cardiovascular Medicine, Radcliffe Department of Medicine, British Heart Foundation Centre of Research Excellence and Wellcome Trust Centre for Human Genetics, University of Oxford, Oxford, United Kingdom; University of Louisville, United States of America

## Abstract

**Background:**

Reduced levels of creatine and total adenine nucleotides (sum of ATP, ADP and AMP) are hallmarks of chronic heart failure and restoring these pools is predicted to be beneficial by maintaining the diseased heart in a more favourable energy state. Ribose supplementation is thought to support both salvage and re-synthesis of adenine nucleotides by bypassing the rate-limiting step. We therefore tested whether ribose would be beneficial in chronic heart failure in control mice and in mice with elevated myocardial creatine due to overexpression of the creatine transporter (CrT-OE).

**Methods and Results:**

Four groups were studied: sham; myocardial infarction (MI); MI+ribose; MI+CrT-OE+ribose. In a pilot study, ribose given in drinking water was bioavailable, resulting in a two-fold increase in myocardial ribose-5-phosphate levels. However, 8 weeks post-surgery, total adenine nucleotide (TAN) pool was decreased to a similar amount (8–14%) in all infarcted groups irrespective of the treatment received. All infarcted groups also presented with a similar and substantial degree of left ventricular (LV) dysfunction (3-fold reduction in ejection fraction) and LV hypertrophy (32–47% increased mass). Ejection fraction closely correlated with infarct size independently of treatment (r^2^ = 0.63, p<0.0001), but did not correlate with myocardial creatine or TAN levels.

**Conclusion:**

Elevating myocardial ribose and creatine levels failed to maintain TAN pool or improve post-infarction LV remodeling and function. This suggests that ribose is not rate-limiting for purine nucleotide biosynthesis in the chronically failing mouse heart and that alternative strategies to preserve TAN pool should be investigated.

## Introduction

Multiple lines of evidence suggest that energy starvation may play an important role in the pathophysiology of heart failure [1], [2]. For example, in the failing heart there is a slow but steady decline in cellular ATP and in total adenine nucleotides (TAN), i.e. the sum of ATP, ADP and AMP, which closely correlates with disease progression [3]. Preservation of TAN has therefore been proposed as a strategy to maintain myocardial [ATP] and thereby function [4].

Adenine nucleotides are too polar to cross the plasma membrane, so loss is a consequence of conversion to nucleosides, e.g. during ischaemia and heart failure, elevated AMP provides increased substrate for 5′-nucleotidase resulting in formation of adenosine, which as a nucleoside, can be lost from the cell by diffusion [5]. Some of this loss will be countered by nucleotide salvage pathways, but TAN pool is also replenished by continuous low-level *de novo* purine synthesis [6]. Phosphoribosylpyrophosphate (PRPP) is the common substrate for both these pathways and is generated from ribose-5-phosphate via the action of PRPP synthetase [7], [8]. Under certain conditions, ribose-5-phosphate availability via the pentose phosphate pathway can be limiting, and this can be circumvented by administration of exogenous D-ribose [9]–[11].

For example, in acute ischaemia there is a rapid and profound loss of TAN pool. Since adenine nucleotides (particularly ADP) inhibit activity of PRPP synthetase [7], this loss of TAN acts to stimulate *de novo* purine synthesis, increasing demand for ribose-5-phosphate. In this context, D-ribose supplementation increases the rate of adenine nucleotide synthesis up to 6 fold in rat, guinea-pig and dog heart; attenuating the decrease in [ATP] and accelerating functional recovery [10], [12]–[16]. However, re-synthesis of adenine nucleotides still takes days [9], [14], and remained too low to significantly increase ATP levels after 3 hours of reperfusion in a dog model [17].

It is not known whether ribose supplementation might also prevent the gradual decline in TAN pool observed in chronic left ventricular (LV) failure. Most notable is a study that gave oral D-ribose with folic acid (a co-factor) to rats with monocrotaline-induced right ventricular dysfunction and showed preservation of TAN pool, reduced fibrosis and diastolic dysfunction after 4 weeks [10]. Clinical studies have been small-scale, short duration, and poorly controlled, but suggest a modest improvement in cardiac function and/or quality of life in heart failure patients treated with ribose [11], [18]–[21]. Thus, the evidence base for chronic ribose treatment in LV failure is poorly established and long-term studies in relevant animal models are missing.

Further evidence suggests synergy when ribose treatment is combined with creatine. This combination was shown to reduce cardiomyocyte apoptosis in an *in vitro* model of ischaemia that was not observed for either agent alone [22]. Recently, we have shown that mice with elevated myocardial creatine are protected from ischaemia-reperfusion injury, but not from chronic heart failure [23]. Notably, this latter result was predicted by an *in silico* study, that went on to hypothesise that a concomitant increase in TAN pool, total exchangeable phosphates (TEP) and creatine levels was necessary to maintain chemical energy in the failing heart [24]. The aim of our study was therefore to test this hypothesis and determine whether a concomitant increase in myocardial creatine and ribose levels would have a beneficial effect on cardiac function and LV remodelling in chronically infarcted mouse hearts.

## Materials and Methods

### Ethics Statement

This study was carried out under Home Office project licence 30/2754, which was approved by the committee for Animal Care and Ethical Review at the University of Oxford, and conforms to UK Home Office Guidance on the Operation of the Animals (Scientific Procedures) Act, 1986. All surgery was performed under full general anaesthesia using isoflurane with post-operative opioid analgesia provided as standard.

### Animal Husbandry

Transgenic mice over-expressing the creatine transporter (CrT-OE) have previously been created in our laboratory and backcrossed to C57BL/6J for more than 10 generations [25]. Two strains were generated exhibiting a wide range of creatine levels from wild-type (WT) concentrations to a >4-fold increase due to variability in transgene mRNA expression. The Tg46 strain was used for this study as its distribution of creatine levels is closer to the target range (88–142 nmol/mg protein, i.e. 20–100% above wildtype). Control groups consisted of a random mixture of WT littermates from CrT-OE mice and C57BL/6J obtained from a commercial breeder (Harlan, UK). Mice were kept in a specific pathogen free environment, with controlled humidity and temperature (20°C to 22°C) and 12 hours light-dark cycle.

### Pilot Study 1: TAN Pool in the Failing Mouse Heart

Female adult C57BL/6J mice underwent coronary artery ligation or sham surgery (n = 7 in each group). Four weeks following surgery, TAN pool was measured in homogenates containing entire left and right ventricles (including scar tissue) using HPLC as previously described [26].

### Pilot Study 2: Bioavailabilty of Ribose in Drinking Water

A 10% (w/v) solution of D-ribose (Sigma-Aldrich) in drinking water was chosen based on the oral dose for human patients and rats (0.15–0.8 g/kg/d) [10], [11], [18], [21], and was shown not to affect water and food intake. Five healthy C57BL/6J mice were treated for 7 weeks, at which time cardiac function was measured by left ventricular catheterisation and compared to control non-treated mice. Animals were killed by cervical dislocation and the heart freeze-clamped for measurement of myocardial ribose-5-phosphate content (Ribose-5-P). Tissue was pulverized into fine powder and protein extracted with ice-cold 10% trichloracetoacetate acid. Tissue debris was sedimented by centrifuging at 13500 rpm for 5 min, 4°C. Resulting supernatant was neutralized with 1 M NaHCO_3_. De-proteinised and neutralized LV extract and 32 units GAPDH were mixed with assay mixture [(in mM):162 imidazole pH 7.6, 30 NAD, 18 MgCl_2_, 15 sodium arsenate, 0.25 erythrose 4-phosphate and 0.03% thiamine pyrophosphate] in a cuvette and incubated for 3 min at 25°C. The first reaction was initiated by addition of 1.25 U of transketolase and absorbance (A_1_) of NADH followed at 340 nm, 25°C for 10 min. A_1_ originates from the presence of glyceraldehyde-3-phosphate and xylulose-5-phosphate in the extract. After the first reaction reached completion, 5 units of ribulose-5-phosphate 3-epimerase and 10 units of phosphoriboisomerase were added to the cuvette. The absorbance (A_2_) was recorded at 340 nm, 25°C for 10 min. Ribose-5-P content of ventricular tissue was expressed as nmol/g wet tissue weight.

### Chronic Heart Failure Study

Female adult mice (21–30 g) were randomly allocated to 4 experimental groups:

- Group **S** consisted of **WT** mice, which underwent **sham** surgery and were not fed ribose;
- Group **MI** consisted of **WT** mice, which underwent myocardial infarction (**MI**) surgery and were given normal drinking water;
- Group **MI+R** consisted of **WT** mice, which underwent **MI** surgery and were given **ribose** in drinking water (10% (w/v) D-ribose solution);
- Group **MI+R+C** consisted of **CrT-OE** mice, which underwent **MI** surgery and were given **ribose** in drinking water (10% (w/v) D-ribose solution).

CrT-OE mice underwent ^1^H-MRS examination to pre-select animals with moderately elevated myocardial creatine (88–142 nmol/mg prot). At least one week later, WT and CrT-OE mice had coronary artery ligation surgery to induce chronic myocardial infarction as previously described in detail by Lygate [27]. Mice received 0.27 mg of buprenorphine subcutaneously for pain relief and ribose supplementation was started 24 hours post-surgery. An echocardiogram was performed ∼4 weeks following surgery under isoflurane anaesthesia using a Visualsonics Vevo2100 to exclude animals with very small or no infarcts. Eight weeks after surgery, surviving mice had cine-MRI to measure infarct size and assess LV remodelling. One week later, haemodynamic function was assessed by LV catheterisation under isoflurane anaesthesia in closed-chest, spontaneously breathing mice using a mikro-tip pressure catheter (SPR-839, Millar instruments, Houston, Texas) as previously described [28]. Measurements were taken at baseline and following maximal stimulation with 16 ng/gBW/min dobutamine. Hearts were excised, rinsed in ice-cold heparinised saline then whole hearts freeze-clamped in liquid nitrogen for measurement of creatine and high-energy phosphates by HPLC [26].

Mice with infarct size below 25% of LV circumference were excluded as these animals do not develop the hallmarks of heart failure [27]. At the end of the study, some mice were excluded in order to retrospectively match groups for the same range of infarct sizes. This is essential to allow inter-group comparison of LV function and remodelling parameters, which are highly dependent on the extent of myocardial injury.

### *In vivo* Magnetic Resonance

All experiments were carried out on a 9.4 T (400 MHz) MR system (Agilent Technologies) using a quadrature-driven birdcage resonator (Rapid Biomedical). For high-resolution magnetic resonance cine imaging (cine MRI), 8–10 short-axis slices, covering the heart from base to apex, were acquired using a cardiac-triggered and respiration-gated fast low-angle-shot sequence with the following parameters: field of view (25.6 mm)^2^, matrix size 256×256, echo time (TE)/repetition time TR) = 1.79/4.6 ms, 15° sinc excitation pulse, number of averages (NA) = 2.

Cardiac ^1^H-MRS was performed as previously described [29]. Briefly, three water suppressed (NA = 256, on-resonance on creatine) and three non water-suppressed scans (NA = 16, on-resonance on water) were acquired interleaved during diastole from a 2 µL septal voxel using a cardiac-triggered/respiratory-gated PRESS sequence (TE/TR = 8 ms/2s). Spectra were quantified using AMARES algorithm from the jMRUI software. Creatine peak amplitudes were normalized to the amplitude of the water peak of the non-suppressed spectra acquired immediately before.

### Statistical Analysis

All data are expressed as mean ± standard deviation. Cine-MRI, haemodynamic and HPLC data were analysed blinded to treatment group. Comparison between two groups was by Student’s t-test. To compare four groups, a one-way ANOVA with Bonferroni’s post-hoc test was used. Linear regression was used to assess the relationship between variables. A chi-square test was performed to assess survival. Differences were considered significant when p<0.05.

## Results

### Pilot Study 1: Adenine Nucleotides are Depleted in the Failing Mouse Heart

TAN pool was measured by HPLC in the remote myocardium of sham-operated and chronically infarcted mouse hearts (n = 7 of each) and was found to be 32.4±2.2 and 27.9±3.6 nmol/mg protein respectively (p = 0.017). This confirmed that TAN pool is reduced by 14% following chronic infarction in the mouse, making it a suitable model to study the effects of ribose supplementation.

### Pilot Study 2: Ribose in Drinking Water is Safe and Bioavailable

**Figure 1:**
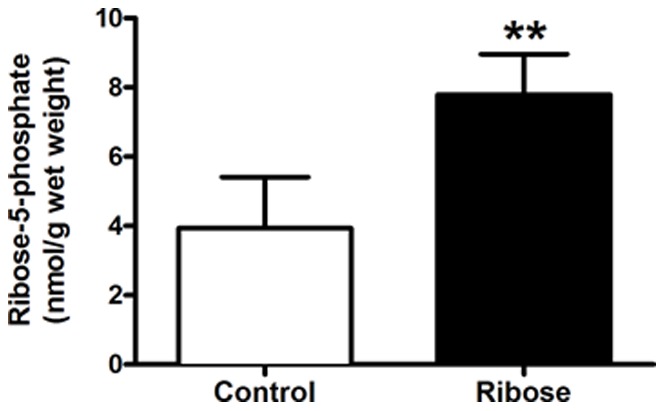
Oral ribose treatment increases ribose-5-phosphate levels in the heart. Myocardial ribose-5-phosphate levels following administration of ribose (10% w/v) in drinking water for seven weeks. Control n = 5, ribose n = 4, mean ± SD, ** denotes p<0.01.

Ribose 10% (w/v) in drinking water was palatable with no change in food and water intake or in body weight, representing an average daily intake of ∼1.5 g/kg of ribose per mouse. Treatment for seven weeks resulted in a 2-fold increase in ribose-5-P levels compared to matched controls receiving pure water (p = 0.004, Figure 1). This had no effect on cardiac function at baseline and following dobutamine stimulation in these healthy mice (stimulated dP/dt_max_ = 12344±2904 (n = 4) vs 11623±4302 mmHg/s (n = 3)).

### Chronic Heart Failure Study: Survival and Fate of Mice Entering the Study

**Figure 2:**
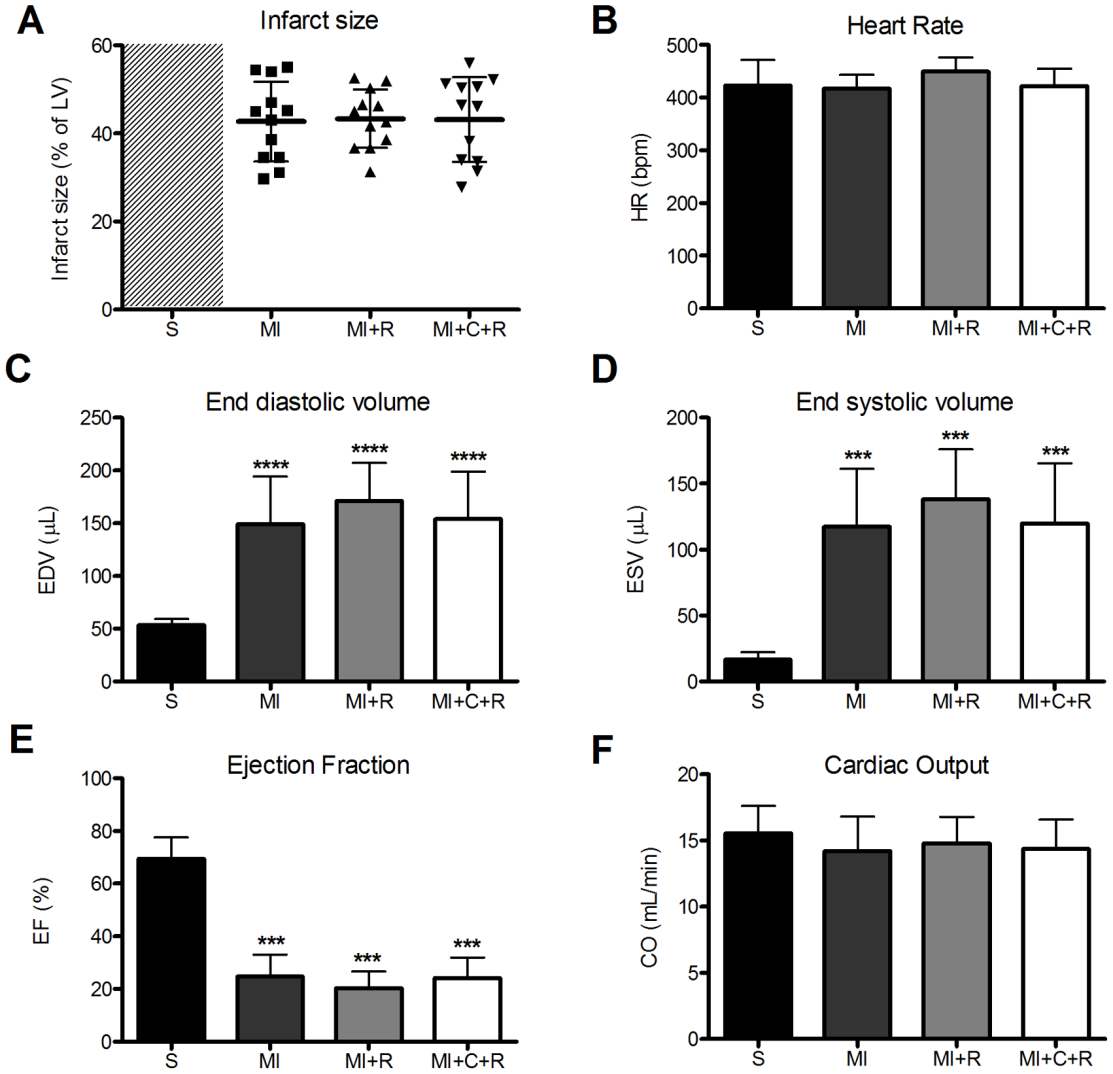
Left ventricular morphology and function derived from MRI 8 weeks post myocardial infarction. Group S are untreated wild-type sham-operated mice; Group MI are untreated wild-type infarcted mice; Group MI+R are wild-type infarcted mice treated with ribose; Group MI+C+R are infarcted creatine transporter overexpressing mice treated with ribose. Infarcted groups were matched for infarct size (A). Left ventricular remodelling and function was measured by cine-MRI (B–F). Data are reported as mean ± SD. *** denotes p<0.001 (1-way ANOVA with Bonferroni’s correction).

The outcome for all mice is summarised in Table 1. LV creatine levels were measured by ^1^H-MRS in 57 CrT-OE mice. Forty-nine mice were within the range of interest (88–142 nmol/mg protein) and underwent myocardial infarction. There was no difference in peri-surgical mortality (during or within 24 hours of surgery) between WT and CrT-OE strains (p = 0.54). Similarly, there was no difference in long-term mortality (24 hours to 8 weeks) between the four study groups (p = 0.43). At the end of the study, mice were retrospectively matched for infarct size (Figure 2A).

**Table 1:** Fate of all mice entering the study.

	WT			CrT-OE
Number of mice entering study for ^1^H-MRS scan	N/A			57
−excluded as outwith target [creatine] range	N/A			8
Number of mice undergoing infarct surgery	92			49
−peri-surgical death (within 24 hours)	18			10
	Sham	MI	MI	MI
	−ribose	−ribose	+ ribose	creatine+ribose
Number of mice at start of ribose feeding	20	27	45	39
−died/euthanised 24 hours to 8 weeks	0	0	4	4
−died during MRI	2	1	3	2
−excluded due to incomplete data sets	0	0	0	2
−excluded due to small infarcts (<25%)	0	7	20	14
−died during haemodynamics	2	4	4	5
Number of mice completing all aspects of study	16	15	14	12
−excluded to match for infarct size	0	3	2	0
Number of mice in final analysis	16	12	12	12

### Chronic Heart Failure Study: Augmenting Myocardial Ribose and Creatine Levels does not Improve Cardiac Function in the Failing Heart

All infarcted groups exhibited cardiac hypertrophy with significant elevation in LV mass compared to sham despite the loss of tissue to infarct scar (Table 2). There was a clear trend for increased lung weight/BW ratio in all groups, suggesting pulmonary oedema commensurate with congestive heart failure (Table 2).

**Table 2:** Morphometry and myocardial biochemistry 8 weeks after myocardial infarction.

	S	MI	MI+R	MI+C+R
	(n = 16)	(n = 12)	(n = 12)	(n = 12)
Body weight (g)	25±3	24±2	25±2	24±2
LV weight (mg) by MRI	79±9	104±9***	116±11*** ^,#^	104±14***
LV/BW (×10^3^)	3.1±0.4	4.3±0.4***	4.6±0.5***	4.4±2.5***
Lung weight/BW (×10^3^)	5.7±0.9	7.0±2.2	7.3±2.4	8.1±2.5*
Total Creatine (nmol/mg prot)	80±6	68±6**	70±9*	108±14*** ^,^ ###
Total adenine nucleotides (nmol/mg prot)	46±7	42±6	42±4	40±3*

All values are mean ± SD. Comparisons were made by one-way ANOVA with Bonferroni’s post-hoc test.

* denotes p<0.05,

** p<0.01,

*** p<0.001 vs group S and ^#^p<0.05,

### p<0.001 vs group MI.

Cardiac dysfunction was noted in all infarcted groups with increased diastolic and systolic ventricular volumes and significantly impaired ejection fraction compared to sham animals (25±8% in group MI, 20±6% in group MI+R, 24±8% in group MI+C+R and 69±8% in group S, p<0.001 for all three infarcted groups versus sham). Cardiac output was maintained to similar levels as in non-infarcted mice (Figure 2), suggesting that LV dilatation was sufficient to maintain stroke volume despite significantly impaired systolic function.

**Figure 3:**
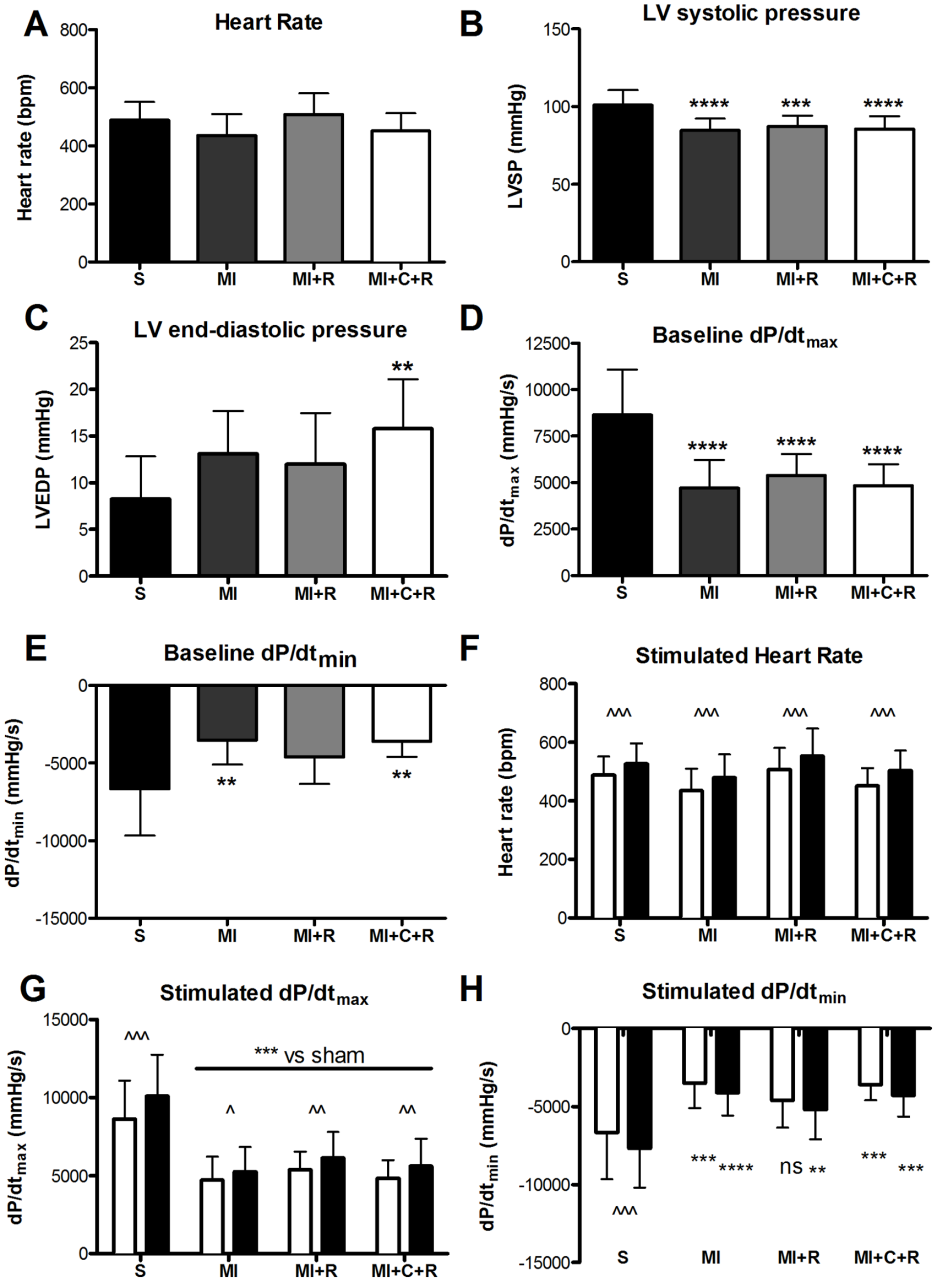
Left ventricular haemodynamic parameters 8 weeks post myocardial infarction. Group S are untreated wild-type sham-operated mice; Group MI are untreated wild-type infarcted mice; Group MI+R are wild-type infarcted mice treated with ribose; Group MI+C+R are infarcted creatine transporter overexpressing mice treated with ribose. Heart rate (A), LV end-systolic and end-diastolic pressures (B–C) and maximal and minimal rates of pressure change (D–E) under baseline non-stimulated conditions. Differences analysed by one-way ANOVA with Bonferroni’s correction. ** denotes p<0.01 and *** p<0.001 versus group S. There was no difference between any of the infarcted groups. Panels F–H show heart rate and maximal and minimal rates of pressure change before and after stimulation with dobutamine (16 ng/g BW/min). Effect of genotype and dobutamine assessed by two-way ANOVA with post-hoc Bonferroni’s correction. ^ denotes p<0.05, ^^ p<0.01, ^^^ p<0.001 for dobutamine (black bars) vs baseline (white bars) and * denotes p<0.05, ** p<0.01, *** p<0.001 versus sham at the same dobutamine dose. Data are mean ± SD.

These findings were confirmed by the invasive assessment of cardiac function both at baseline and following dobutamine stimulation. At baseline, heart rate was comparable between all groups indicating a similar anaesthetic depth. Left Ventricular Systolic Pressure (LVSP) was lower and Left Ventricular End Diastolic Pressure (LVEDP) elevated in the infarcted groups (Figure 3B and 3C). A reduction in the inotropic (decrease in dP/dt_max_) and lusitropic (decrease in |dP/dt_min_|) properties of the heart was observed (Figure 3D and 3E). All these changes are characteristic of the failing heart, but there were no differences between treatment and non-treatment groups. Beta-adrenergic stimulation with dobutamine increased dP/dt_max_ in all groups, but was more pronounced in sham than infarcted groups, indicating impaired contractile reserve. Again no difference was observed between infarcted groups (Figure 3F–H).

### Chronic Heart Failure Study: Total Creatine and Adenine Nucleotide Pools

Individual phosphocreatine, ATP, ADP and AMP levels cannot be reliably measured in the mouse heart in this experimental model, since measurements mainly reflect degradation during sample collection and preparation. However, the sum of these components are biochemically meaningful. Total creatine levels were lower by 15% in MI mice compared to sham (p<0.01) (Table 2) with a similar 13% reduction observed in group MI+R (p<0.05). However, infarcted CrT-OE mice maintained significantly higher creatine levels compared to sham (p<0.001) despite a 10% decrease in myocardial creatine concentration with development of heart failure (p<0.05). In all 3 infarcted groups, TAN pool was decreased by 8 to 12% (and with no significant difference among infarcted groups), but compared to sham, this only reached statistical significance in group MI+C+R (Table 2).

**Figure 4:**
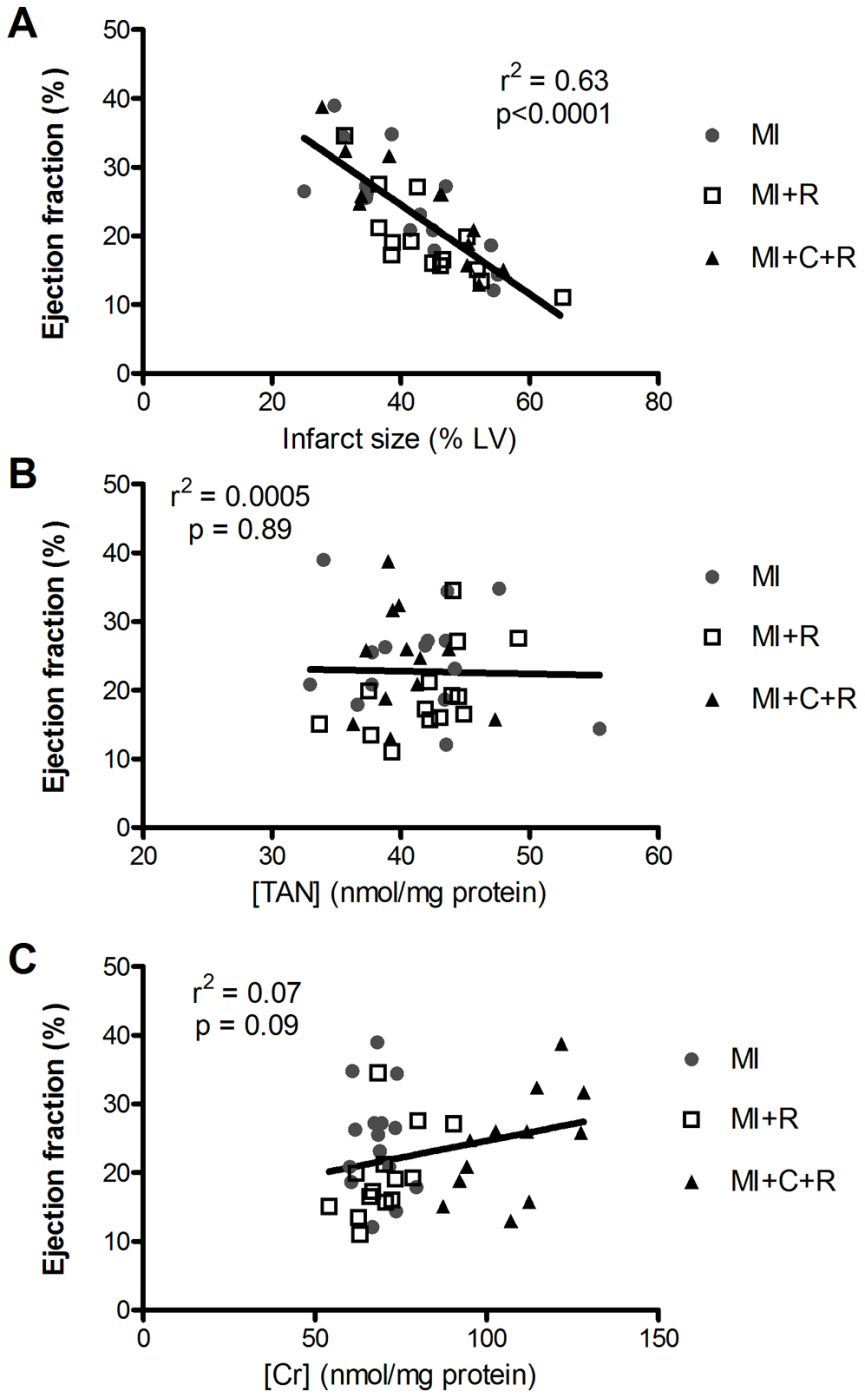
Factors influencing ejection fraction by correlation analysis. Ejection fraction assessed by MRI 8 weeks after myocardial infarction correlated well with infarct size (A), but not with myocardial total adenine nucleotides (B), or myocardial creatine levels (C). Correlation analysis and linear regression is for all groups analysed together. Group MI are untreated wild-type infarcted mice; Group MI+R are wild-type infarcted mice treated with ribose; Group MI+C+R are infarcted creatine transporter overexpressing mice treated with ribose.

There was no relationship between LV TAN or total creatine levels and ejection fraction in the infarcted mice (Figure 4). In contrast, a strong correlation between infarct size and ejection fraction was observed irrespective of genotype or ribose treatment (r^2^ = 0.63, p<0.0001).

## Discussion

This is the first study to assess the effect of ribose administration in chronic left-sided heart failure. We show that oral ribose is palatable and bioavailable and that reduced TAN pool is a feature of murine heart failure, albeit to a lesser extent than in larger mammals. However, ribose treatment was ineffective at preventing this decline, and neither ribose on its own, nor in combination with elevated creatine, altered the pattern of adverse cardiac remodelling and LV dysfunction characteristic of the failing heart.

### Validation of Ribose and Creatine Elevation in the Myocardium

We demonstrated that ribose administered in drinking water was well tolerated by mice and induced a 2-fold increase in ribose-5-P concentration in the myocardium. This confirms that ribose is bioavailable when given orally in the mouse. The concentration of ribose observed in control hearts was in agreement with the published literature for rat hearts [30]. However, to our knowledge this is the first time that ribose supplementation has been shown to elevate myocardial tissue levels, which represents a major limitation of the published literature to date.

Similarly, we confirmed that over-expression of the creatine transporter resulted in elevated myocardial creatine levels, and these were maintained supra-physiological following infarction despite a decrease of 10% from starting values. The levels obtained are within the range recently shown to be beneficial in ischaemia-reperfusion injury [23], suggesting this is an appropriate dose for therapeutic effect without the adverse effects associated with very high creatine levels [25]. We have previously demonstrated that there is no benefit of creatine elevation alone [23], and have now shown that combining elevated creatine with ribose has neither beneficial nor deleterious effects in this model of chronic heart failure.

### Influence of Species on Adenine Nucleotides and Creatine in the Failing Heart

In the infarcted mouse heart, we observed a consistent decrease in TAN pool by 8–14%. This is a small change compared to other species and therefore did not always reach significance. Decreases of up to 50–60% have been measured in the failing rat heart [10] and in patients with end-stage heart failure at the time of cardiac transplant [9], while in the dog pacing model the decrease in TAN closely followed progression of heart failure to reach 21% after 8 weeks [3]. This has similarities to the modest reduction in creatine levels in the failing mouse heart, 11–16% compared to 25–56% commonly observed in humans and other species [31]. In this context it could be argued that the mouse is not the ideal species in which to study these effects. However, key parameters such as maximal velocity of the creatine kinase reaction still correlate with disease severity in the mouse despite such modest changes [31]. In our study, creatine+ribose supplementation could not prevent the significant 13% fall in TAN pool, but we cannot rule out that this approach might have been effective in attenuating a much larger fall in other species.

### Why did Ribose Supplementation not Maintain TAN Pool in the Failing Heart?

Previous studies have shown beneficial effects in acute models of ischaemia where a large and rapid drop in TAN pool occurs as a consequence of nucleotide depletion and subsequent adenosine release [14]–[16]. This is very different to the slow gradual decline observed in non-ischaemic myocardium in the failing heart, for which the mechanisms are still unclear. One possibility is that such gradual loss is insufficient to stimulate adenine nucleotide salvage pathways. It has also been suggested that *de novo* purine synthesis is inhibited in the failing heart [3], in which case providing excess ribose would only have limited effect. Another explanation is that the severe loss of TAN in ischaemia removes end-product inhibition of the purine synthesis pathway [7], but that the modest loss of TAN pool observed in our heart failure model was simply insufficient to stimulate *de novo* purine synthesis, which meant that ribose levels were not limiting. For this reason, ribose supplementation may paradoxically be effective in models with the largest reductions in TAN pool.

### Study Limitations

Following the above argument, we cannot rule out that a longer duration of heart failure may have resulted in a more robust loss of TAN and more pronounced effects of ribose treatment. It is a limitation of our study that elevated myocardial ribose levels were demonstrated only in healthy hearts, and this was due to the assay requiring tissue from the entire heart. We cannot therefore eliminate the possibility, however unlikely, that ribose uptake is impaired in chronically failing myocardium. Furthermore, in most published studies, ribose was administered prior to the induction of acute dysfunction. This may have brought the myocardium into a more favourable condition to handle the subsequent insult, possibly by activating adenine nucleotide salvage pathways [32]. Finally, we opted not to include a group of CrT-OE without ribose treatment since these mice have already been studied in this heart failure model [23], however this means there is no data on how elevated creatine alone affects TAN pool.

### Augmentation of TAN Pool as a Therapeutic Strategy

This aim of this study was to test the hypothesis arising from *in silico* modelling that combined elevation of creatine, TAN and total exchangeable phosphate pools would be favourable to the failing heart [24]. This hypothesis remains untested, since elevating ribose and creatine levels were not sufficient to preserve TAN pool in our murine model of heart failure. This is in contrast to the effect of ribose in models of acute ischaemia and suggests that ribose is not rate-limiting for *de novo* purine nucleotide synthesis in the failing mouse heart. However, it does not rule out an effect of ribose in heart failure models where the drop in TAN pool is more profound. Other approaches to preserve TAN pool deserve to be tested, and may yet prove beneficial, for example, inhibition of 5′-nucleotidase to prevent nucleotide degradation, modulation of glucose-6-phosphate dehydrogenase as the rate limiting enzyme of the pentose phosphate pathway, up-regulation of adenosine kinase, and nucleotide transporter inhibitors.

### Conclusion

Using a combination of genetic modification and supplementation we elevated ribose and creatine levels in the mouse heart. This did not prevent gradual loss of total adenine nucleotides in remote myocardium following chronic myocardial infarction and did not protect against cardiac remodelling and development of heart failure.
